# In-Bore MRI-Guided Ureteral Stent Placement During Prostate Cancer Cryoablation—A Case Series

**DOI:** 10.3390/diagnostics15141781

**Published:** 2025-07-15

**Authors:** Sydney Whalen, David Woodrum, Scott Thompson, Dan Adamo, Derek Lomas, Lance Mynderse

**Affiliations:** 1 University of Illinois College of Medicine, Chicago, IL 60612, USA; 2 Mayo Clinic Department of Radiology, Rochester, MN 55905, USA; 3 Mayo Clinic Department of Urology, Rochester, MN 55905, USA

**Keywords:** prostate cancer, cryoablation, ureteral stent, interventional MRI

## Abstract

**Introduction**: Ureteral stents are widely used in the specialty of urology to preserve renal function and provide ureteral patency in cases of urolithiasis, strictures, malignancy, and trauma. This paper presents a novel application of prophylactic ureteral stents deployed under MRI-guidance for ureteral protection in the setting of in-bore salvage cryoablation therapy for recurrent and metastatic prostate cancer. This is the first known case series of ureteral stent placement using near real-time MRI. **Materials and Methods**: A retrospective chart review was performed for all patients who underwent MRI-guided ureteral stent placement prior to in-bore cryoablation therapy from 2021 to 2022. Each case was managed by an interdisciplinary team of urologists and interventional radiologists. Preoperative and postoperative data were collected for descriptive analysis. Physics safety testing was conducted on the cystoscope and viewing apparatus prior to its implementation for stent deployment. **Results**: A total of seven males, mean age 73.4 years (range 65–81), underwent successful prophylactic, cystoscopic MRI-guided ureteral stent placement prior to cryoablation therapy of their prostate cancer. No intraoperative complications occurred. A Grade 2 postoperative complication of pyelonephritis and gross hematuria following stent removal occurred in one case. The majority of patients were discharged the same day as their procedure. **Conclusions**: This case series demonstrates the feasibility of in-bore cystoscopic aided MRI guidance for ureteral stent placement. Ureteral stents can be used to increase the safety margin of complex cryoablation treatments close to the ureter. Furthermore, by following the meticulous MRI safety protocols established by MRI facility safety design guidelines, MRI conditional tools can aid therapy in the burgeoning interventional MRI space.

## 1. Introduction

Ureteral stents are an indispensable tool for maintaining patency of the ureters in the setting of obstruction or injury. The earliest stents were self-manufactured from silicone for patients with ureteral strictures, ureterovaginal fistula, or malignant obstruction [1,2]. They have since been adapted to aid ureteral identification during laparoscopic surgery and to preserve renal function in procedures where ureteral damage/narrowing is a concern [3]. Therefore, the placement of ureteral stents can occur as a prophylactic measure prior to surgical intervention or in response to an obstruction, often caused by renal stones, adhesions, or malignancies [4].

Regardless of the indication, the procedure to place ureteral stents has not changed significantly since their mass-adoption nearly half a century ago. First, a cystoscope is placed transurethral into the bladder and a guidewire is advanced into the ureter. A measuring ureteral catheter is placed over the wire into the renal pelvis, typically with fluoroscopic guidance based on expected stent length. Deployment of the stent can be conducted blindly or with image guidance to confirm the final position.

Advances in MRI technology have significantly improved contrast resolution and signal to noise ratio, paving the way for diagnostic and interventional applications under MRI-guidance [5]. Limitations to their development include MRI-compatibility issues of the cystoscope and associated hardware [6]. Despite these obstacles, ablation devices have evolved MRI-safe ablation needles for cryoablation, laser, microwave ablation, and focused ultrasound, and these ablation procedures have proven safe and effective for prostate cancer and vascular malformations [7,8]. MRI-guided cryoablation has been demonstrated to be an effective tool to target prostate cancer recurrences in the prostate bed and pelvis [9]. As such, this requires protection of vital structures close to the ablation target and adjacent to the ablation zone, specifically the ureters, urethra, and rectum. We present a case series where near real-time MRI imaging was used for ureteral stent placement as a prophylactic measure before cryoablation therapy for prostate cancer. This concurrent anesthesia approach obviates the need for a separate procedure under anesthesia using fluoroscopy or CT guidance.

## 2. Methods

All patients who underwent in-bore MRI-guided ureteral stent placement prior to cryoablation for recurrent or metastatic prostate cancer between January 2021 and February 2022 were reviewed. Each case was managed by an interdisciplinary team of urologists, interventional radiologists, anesthesiologists, MRI physicists, and technicians who specialize in MRI interventional procedures. Pre- and postoperative data were collected for descriptive analysis.

### 2.1. Indications for Ureteral Stent Placement During MRI-Guided Cryoablation

Prophylactic ureteral stent placement is indicated when cryoablation poses a significant threat to the ureter. In the setting of a rising prostate-specific antigen (PSA), recurrent and metastatic prostate cancer can first be localized using multi-parametric MRI or 11-C Choline/PSMA PET imaging [10,11]. If a recurrent prostate cancer lesion is found near the ureter, stent placement can be used to protect the ureter from procedural edema or injury from the treatment ice ball and provide a potential framework for healing if the ablation zone encompasses the ureter. The stent provides a means of maintaining kidney drainage should an unintentional injury or narrowing secondary to edema occur. One of the most common places for recurrent prostate cancer is within the seminal vesicles, which are inherently close to the ureters. Therefore, when planning for MRI-guided cryoablation of a seminal vesicle recurrence, it was necessary to have ureteral stent placement. Although ureteral stents can be placed prior to the ablation, this necessitates two procedures and two different sedations for the patient. The ability to provide ureteral stent placement within the MRI at the same time as the MRI-guided cryoablation streamlines the procedure for the physician and the patient.

The appropriate patients for peri-operative ureteral stent procedure are those with proximity of the ablation zone ice ball to the adjacent ureter with potential for ureteral wall injury/edema. In this series, all patients had alterations in their anatomy due to recurrences after prior therapy for prostate cancer (e.g., surgery, radiation, chemotherapy, or androgen deprivation). Alternative treatment approaches were offered if the tumor ablation were to totally encompass the ureter and result in complete ureteral obstruction (i.e., urinary diversion or nephrostomy drainage). In this consecutive series of patients, no stent placement failure was encountered.

MRI guidance for ureteral stent placement provides a novel imaging platform for stent deployment immediately prior to, or concurrent with, MRI-guided cryoablation treatment.

### 2.2. Pre-Procedural MRI Physics Safety Testing

**Figure 1:**
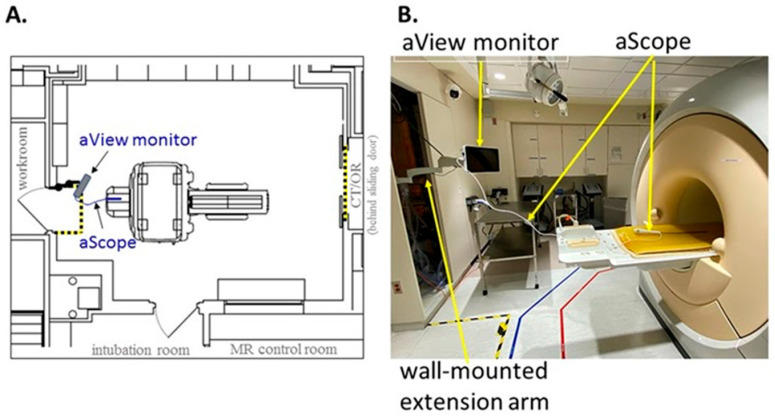
(**A**) Floorplan outlining Ambu^®^ aView™ Advance HD monitor and Ambu^®^ aScope™ cysto single-use cystoscope within MRI suite. (**B**) Positioning of Ambu^®^ system with maximal extension of the wall-mounted arm relative to the MRI scanner.

Due to the inherent dangers associated with the magnetic field and radiofrequency waves, new equipment must be tested and approved by a designated safety committee before use in patient care. A report was submitted to the MRI safety committee for Ambu^®^ aView™ Advance HD monitor and Ambu^®^ aScope™ single-use cystoscope. The report contained information on device positioning relative to the MRI scanner (Figure 1), tethering, Zone 4 deflection tests, and impact on MRI image quality. The report suggests the Ambu^®^ aScope™ single-use cystoscope contains ferrous components following a hand-held magnet test outside of Zone 4. The device poses negligible projectile risk in clinical practice supported by multiple drop tests from the bore edge in which the device falls under its own weight and travels a maximum distance of 25 cm into the bore. As such, the devices were conditionally approved by our MRI Safety Board for MRI-guided ureteral stent placement with the MRI environment based on a detailed analysis and instruction for use at our institution.

### 2.3. Ureteral Stent Placement Procedure

**Figure 2:**
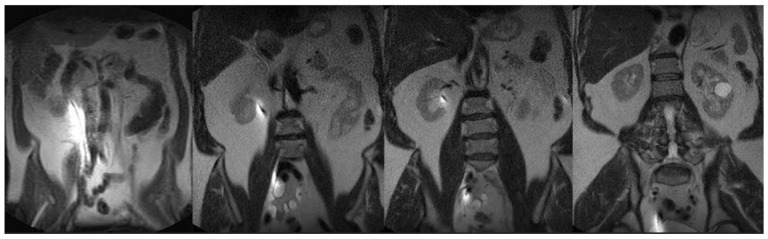
Coronal FSE T2 weighted 45 s scan (FOV 400, TE 80 ms/TR 100 ms) with the nitinol wire susceptibility artifact from renal pelvis to bladder prior to advancing the ‘double J’ ureteral stent.

**Figure 3:**
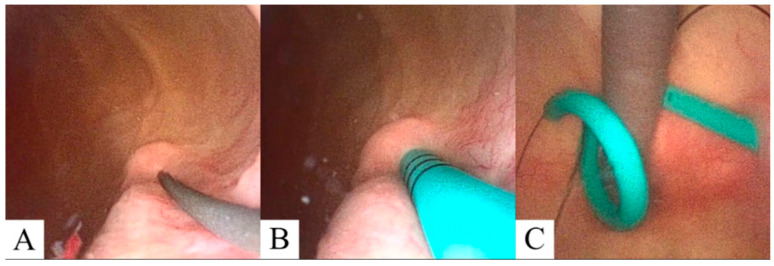
Cystoscopic visualization of the guidewire (**A**) and ureteral stent (**B**) projecting from the ureteral orifice. (**C**) Retroflex cystoscopic visualization showing cystoscope (gray) and distal ureteral stent (cyan) curl prior cryoablation procedure with attached retrieval ‘dangle’ (black).

Anesthesia is initiated in the MRI (Philips 1.5 T Ingenia, Amsterdam, The Netherlands) anteroom adjacent to the MRI suite. The patient is transferred to the scanner and positioned supine on the MRI table in a semi-frog-leg position, sterilely prepped and draped with access from the back of the magnet. A single-use flexible cystoscope (Ambu aScope 4, Ambu, Inc., Columbia, MD, USA) is placed per urethra at the MRI bore back entrance, and a straight 0.035 inch nitinol wire is passed through the appropriate ureteral orifice to the kidney until gentle resistance is met. Next, a standard 6 French open ended, graduated length ureteral stent is utilized to measure the ureteral length. At this point, MRI balanced FSE T2 coronal imaging is performed to visualize wire position within the ureter and renal pelvis (Figure 2). After confirmation, an appropriately sized double-J ureteral stent is advanced over the wire alongside the cystoscope with direct cystoscopic observation of the ureteral orifice. A balanced FSE T2 coronal MRI image is obtained to document the definitive location of the stent. A confirmative cystoscopic view of the distal end of the double-J stent is obtained within the bladder (Figure 3). All of the commercially available hydrophilic coated, ‘double J’ ureteral stents made from co-polymers of the silicone family and polyethylene/polyurethane are MRI compatible (Bard Inc., Murray Hill, NJ, USA, Boston Scientific Inc., Marlborough, MA, USA, and Cook Medical Inc., Bloomington, IN, USA). The range of length for this study was 22–26 cm and 6 Fr ‘double J’. The MRI compatible (non-ferromagnetic) hydrophilic coated, nitinol (nickel/titanium alloy) 0.035 in, 150 cm, straight wire was used to cannulate the ureteral orifice cystoscopically (Terumo, Inc., Somerset, NJ, USA).

The average total procedure time for this cystoscopically aided MRI guided ‘in bore’ ureteral stent placement was 18 min. This is a substantial saving from a separate, traditional cystoscopic ureteral stent placement under anesthesia with fluoroscopy or CT guidance and accomplished concurrently with the ablative procedure under the same anesthesia. Granted, this ‘in bore’ cystoscopic procedure was conducted with a veteran MRI interventional team with extensive experience performing a myriad procedures and included an MRI physicist, MRI technologist, Anesthesiologist, Interventional Radiologist, and Urologist. Widespread adoption of this approach would fit well with experienced teams performing interventional MRI.

Following ureteral stent placement, the procedure transitions to the MRI-guided cryoablation of the prostate. An MRI-compatible cryoablation system (Visual ICE MRI, Boston Scientific Inc., Marlborough, MA, USA) and transperineal grid system are used to conduct the ablative therapy, as previously described using a perineal approach [9,12,13]. The iceball is monitored under continuous MRI-guidance and cryoablation margins are noted. Every effort is made to prevent cryoablation of surrounding structures, including the ureters, bladder, urethra, and rectum. If the iceball margin touches the outer edge of the ureter, the stent will be left in place for 6 weeks. In the event that the iceball edge does not touch the outer edge of the ureter, the stent may be removed at the end of the case by means of the attached ‘dangle’ exiting the urethra.

### 2.4. Stent Removal

Well-known complications of ureteral stents include discomfort, encrustation, and bacterial infection, which can be mitigated by timely removal, alpha adrenergic blockade, or anticholinergic oral medications [14]. If adequate cryoablation safety margins are obtainable, ureteral stents may be removed once the procedure is complete.

If ureteral injury or significant post-ablation edema is suspected due to proximity of cryoablation margins, a stent can be left in place to provide urinary drainage, prevent strictures, and promote healing.

## 3. Results

Seven males with a median age of 73.4 years (range 65–81) underwent cystoscopic-assisted prophylactic ureteral stent placement under MRI guidance prior to cryoablation therapy for recurrent or metastatic prostate cancer. Preoperative disease characteristics are summarized in (Table 1).

All patients had unilateral ureteral stent placement to mitigate injury related to cryoablation therapy. A total of five patients (71.4%) underwent treatment for recurrent prostate cancer within the remnant seminal vesicles or the post-surgical seminal vesicle bed structures. Two patients (28.5%) were treated for metastatic disease, one to the bladder neck and another to the pelvic sidewall with obturator lymph node involvement. 

**Figure 4:**
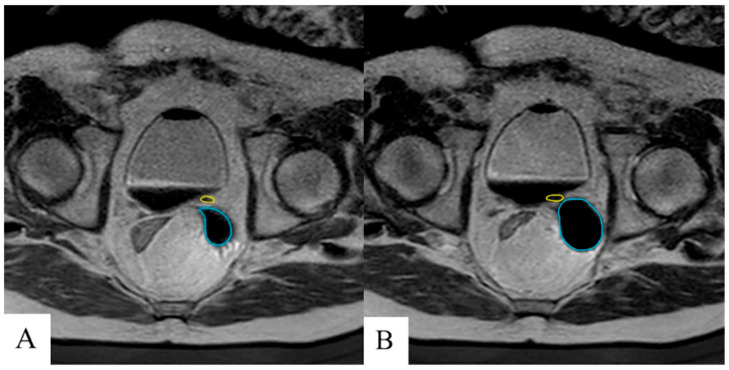
Near real-time T2 MRI to measure iceball growth (cyan) and proximity to the ureter (yellow) during early (**A**) and late (**B**) cryoablation in which a margin of 2 mm between the ureter and cryoablation border was maintained. Ureteral stent was left in place at discharge to maintain patency.

Ureteral stents were removed in five of seven (71.4%) patients prior to discharge due to adequate cryoablation safety margins to the ureter at risk noted on real-time MRI. Two patients (28.5%) were discharged with indwelling ureteral stents because of significant procedural edema or in the event of potential damage to the ureters incurred during cryoablation therapy (Figure 4). The first patient had the indwelling ureteral stent left in place following MRI-guided cryoablation of the left seminal vesicle. The second patient had the indwelling ureteral stent left in place following MRI-guided cryoablation of the vesicourethral anastomosis having had two previous indwelling ureteral stents. In both patients, the indwelling ureteral stents were removed via cystoscopy at thirty-six and forty-nine days post-ablation, respectively, with the latter stent exhibiting mild encrustation with no further complications. The majority of patients were discharged the same day as their procedure (57.1%) with the maximum time to discharge being two days in a single patient with a history of orthostatic hypotension and a risk of falls.

One patient with a known prothrombin gene mutation experienced a Grade 2 (Clavien-Dindo) complication following ureteral stent removal [15]. The stent was removed on the day after their cryoablation procedure. The patient noted gross hematuria within hours of discharge, leading to overnight admission and pharmacologic treatment for pyelonephritis. Four days later, the patient experienced flank pain, gross hematuria, and clots after resuming Xarelto which had been discontinued prior to the cryoablation procedure. No other complications occurred.

## 4. Discussion

Although MRI is not a common choice for procedural guidance, the past several decades have seen an increase in the number of MRI-safe procedural tools and clinical indications for MRI-guided procedures. Superior soft tissue image guidance is the cornerstone of why many choose to use it for successful, minimally invasive interventions. Magnetic resonance imaging provides unique soft tissue conspicuity without ionizing radiation, and the marriage of near real-time multiplanar imaging with superior soft tissue contrast make MRI an excellent option for interventional therapies for the prostate and associated cancer recurrences [16].

MRI-guided cryoablation for prostate cancer has been described as treatment options for patients who had localized recurrences following radical measures [9]. More recently, salvage cryo-ablation has been compared to other localized options like high-intensity focused ultrasound (HIFU) [17]. The efficacy of cryoablation is thought to be comparable, if not superior, though larger cohort studies are required to decipher the benefits of each modality. 

The advantages of MRI-guidance for salvage prostate interventions are magnified in the setting of post-surgical and salvage radiotherapy where subsequent treatment options become limited. MRI provides superior spatial resolution, multi-planar, near real-time monitoring of the ablation zone, and with exquisite visualization of associated normal structures [18]. The ability to determine ablation margins during treatment and at the end using T2 or dynamic contrast imaging is an additional advantage [9].

While ureteral stents have been used in the field of urology for nearly half a century, their use in combination with MRI-guided cryoablation for recurrent or metastatic prostate cancer treatment safety is novel. The physical and technical risks associated with MRI-guided ureteral stent deployment and cryoablation therapy are related to the magnetic field which can have a projectile effect on ferrous objects [19]. MRI-guided procedures can be facilitated by physically locating the MRI-unsafe components outside the MRI procedure room. In our situation, the cystoscope viewing monitor was bolted to the wall and the images transmitted to an MRI safe monitor next to the MRI bore. While ureteral stents have traditionally been manufactured out of silicone which is MRI-safe [1], newer magnet-tipped and metal ureteral stents must be avoided [20].

While the efficacy of prophylactic ureteral stent placement in laparoscopic surgery is disputed [3], their use in CT-guided microwave [21] and cryoablation [22] for renal cell carcinoma is supported in small cohort studies. This is the first known report of cystoscopically aided MRI-guided ureteral stent placement for cryoablation of recurrent and/or metastatic prostate cancer. The procedure has been demonstrated safe and feasible without significant alterations to the methods of stent deployment or cryoablation once appropriate MRI safety testing and unique procedural measures have been satisfied. MRI-guided ureteral stent placement in this setting is advantageous to mitigate radiation exposure, to decrease patient procedures, and to be performed under one anesthetic. Traditionally, prophylactic ureteral stent placement prior to MRI-guided cryoablation therapy was performed in the operating room, requiring the intubated patient to be transferred to the MRI suite, but this approach suffered from OR availability timing and long distance of transporting an anesthetized patient within the hospital [9]. This novel combined procedure for stent deployment minimizes these negative aspects to deliver more precise patient care in a timely fashion.

The single complication associated with this case series using cystoscopically aided MRI-guided ureteral stent placement is not unexpected. In retrospective studies, ureteral stents cause hematuria in 10.4% of patients, which is likely related to urothelial irritation of the renal pelvis, ureter, or bladder [23]. The patient’s known prothrombin gene mutation predisposes him to clotting which could help further explain the adverse response to ureteral irritation. Bladder discomfort and spasm were observed in previous accounts of ureteral stents used for MRI-guided cryoablation [9], though a larger cohort is required to elucidate complication rates of this procedure. This consecutive series of ‘in bore’ MRI assisted cystoscopic placement of ureteral stents in complex recurrent prostate cancer patients is not intended to replace current fluoroscopic placement of ureteral stents, but to demonstrate the facility with which this can be accomplished by an experienced team during the same anesthesia as the cancer ablation in select group of patients, obviating the cost, anesthesia time and additional risk of two separate procedures.

## 5. Conclusions

This is the first known case series to report cystoscopic aided ureteral stent placement using MRI guidance in the setting of recurrent, biopsy-proven prostate cancer treatment with MRI-guided cryoablation. The procedure can be completed safely prior to cryoablation of recurrent or metastatic prostate cancer. This provides a mechanism of urinary drainage if cryoablation margins cannot reliably avoid the ureter. Further studies are necessary to determine the long-term efficacy and complications associated with MRI-guided ureteral stent placement in the burgeoning field of ablative therapies for prostate cancer.

## Figures and Tables

**Table 1:** Demographics and preoperative disease characteristics (Age, BMI, PSA, location of the prostatic or extraprostatic lesion, and relevant treatment history).

Demographics and Disease Characteristics	
Mean Age (years)	73.5
Mean BMI (kg/m^2^)	28.73
Mean PSA (ng/mL)	2.75
Disease Location	
Left	4/7 (57.1%)
Right	3/7 (42.9%)
Disease Involvement	
Seminal Vesicle Remnant or Bed	5/7 (71.4%)
Obturator Lymph Node	1/7 (14.3%)
Adjacent Bladder	1/7 (14.3%)
Prostate Cancer Treatment History	
Prostatectomy	7/7 (100%)
Salvage Pelvic Lymphadenectomy	2/7 (28.5%)
Adjuvant/Salvage Radiation	5/7 (85.7%)
Chemotherapy	2/7 (28.5%)
Androgen-Deprivation	2/7 (28.5%)
Prior Ablation	3/7 (42.9%)

## Data Availability

The raw data supporting the conclusions of this article will be made available by the authors on request.
